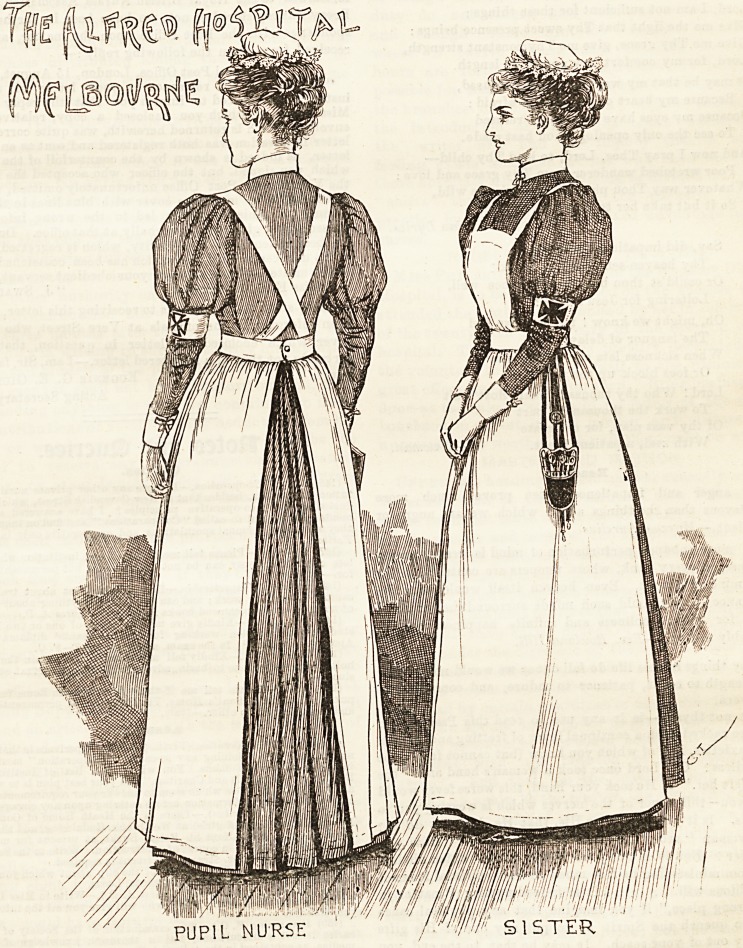# "The Hospital" Nursing Mirror

**Published:** 1896-08-22

**Authors:** 


### "The Hospital" Nursing Mirror.

Being the Extra Nursing Supplement of "The Hospital" Newspaper.

[Contributions for this Supplement should be addressed to the Editor, The Hospital, 428, Strand, London, W.C., and should have the word "Nursing" plainly written in left-hand top corner of the envelope.]

### News from the Nursing World.

### NEW NURSING HOMES IN LONDON.

Three new and up-to-date nursing homes will be ready and open for inspection before long. That at the London Hospital is being rapidly finished, some delay in its completion having been due to the building strike in the early summer. Miss de Pledge informs us that the new nurses' quarters at the Chelsea Infirmary will very soon be ready for occupation; and at the Poplar and Stepney Sick Asylum the last touches are now being given to the recreation-rooms of the new home. The bed-rooms are already in use.

### THE POISON BOTTLE ONCE MORE.

An inquiry was held by Mr. H. R. Oswald on Tuesday last, at the Western Fever Hospital, Seagrave Road, Fulham, respecting the death of a child of fifteen months, to whom a nurse had administered a dose of chlorate of potash in mistake for lime-water. The doctor's evidence showed that the child was dangerously ill with diphtheria when admitted. Post-mortem examination demonstrated that death was due to syncope from diphtheria, and was not, in his opinion, even accelerated by the chlorate. The nurse explained that the lime water was in a green bottle with a white label and the chlorate of potash in a green bottle with a red label. She did not know how she made the mistake. The jury returned a verdict in accordance with the medical evidence, and added a rider hoping that the authorities "would make such an alteration in the kind of bottles used that in future such a mistake could be avoided."

### LOCAL GOVERNMENT BOARD INQUIRIES.

The holding of a Local Government Board inquiry into the management of an institution under its jurisdiction is by no means the simple matter which those who have never been present at one might imagine. Long days are devoted to the consideration of a multitude of petty complaints made by various witnesses, and these are often tinged by personal spite, which is calculated to wreck the future harmony and internal discipline of the establishment whence they emanate. Whether the grievances are wholly or even partially true is overlooked when they have once become public property. The outside world notes and condemns internal discord in a public institution devoted ostensibly to works of charity. The gravity of the position is largely increased by the presence of counsel, who cross-examine the witnesses, and emphasize the partisanship which they are naturally inclined to encourage. The Local Government Board itself is represented by its Medical Inspector, who brings a store of experience to bear on the matters under discussion, and, by tact and discernment, does his best to keep personalities in abeyance. Realising how much has been done through the influence of the Local Government Board for the practical comfort of those who work in infirmaries under the poor law, we cannot but believe that at no distant day they will issue a fresh code of orders for the guidance of their officers. Those now in existence apply rather to the period at which they were framed than to the present day, when matrons are intelligent trained nurses, instead of women promoted from the domestic staff or the wards of the institution. Twenty or thirty years ago the medical officer was presumably the only authority on nursing matters, and he was forced to devote such leisure as he could to instructing ignorant women in elementary tendance of the sick. To-day the advance made in the art of nursing enables the modern doctor to confine his attention to the scientific treatment of disease, leaving the carrying out of his orders to skilled women, to whose province the personal care of the sick properly belongs.

### EXTENSION OF NURSES' QUARTERS AT SUNDERLAND INFIRMARY.

At the recent annual court of governors of the Sunderland Infirmary, the question of increased and improved accommodation for the nursing staff was considered, the committee asking for permission to apply the sum of £1,300 from the funds should subscriptions from the public fall short of the estimated outlay. The Chairman, Mr. James Laing, in moving the adoption of the report, made an urgent appeal to the friends of the infirmary to come forward and help in providing the nursing staff with proper and comfortable quarters. A pleasant spirit of unanimity seemed to reign amongst those present as to the desirability of giving the nurses "healthy rooms" to live in, which will, we trust, result in the speedy raising of the money needed, and its application in the best and wisest way. In their report the medical board expressed special appreciation of "the excellent manner in which the nursing of the infirmary continued to be carried out by the sisters and nurses of the staff." During the past year thirteen probationers out of fifteen passed the examiners in anatomy and surgical nursing, and ten out of twelve in physiology and medical nursing.

### THE HELENA HOME FOR POOR LADIES.

For seventeen years past there has existed in Brownlow Road, Reading, a "Home of Peace," intended for poor gentlewomen suffering from incurable illness, and needing care and skilled nursing during their last months of life It is not an institution for destitute cases, but for the benefit of those who are homeless and without relations, and able only to pay a small sum for maintenance. The home is managed at small cost, the lady-in-charge, Miss Emily Morris, and the medical officers, giving their valued services gratuitously. Outside friends can greatly help such an institution by taking the invalids for drives, or contributing fruit and flowers, and so forth, for their comfort and pleasure, and it is good to know that the Helena Home has several kind benefactors in these respects. Those resident in or near Reading who are not already acquainted with this excellent little home would do well to visit it forthwith, and to give it the support needed to carry on efficiently and satisfactorily a truly good work.

### IN AID OF THE NORTH RIDING NURSING ASSOCIATION.

By permission of the Hon. Admiral and Mrs. Carpenter a very successful garden party was held the other day in Kiplin Park for the benefit of the North Riding Nursing Association. The Marchioness of Zetland is president of the association, and was present herself during the afternoon. Pastoral plays were acted during the day in "the old garden," and proved very popular.

### ANOTHER POOR-LAW VICTIM.

At an inquiry held by the Salford coroner on the death of a patient at the Hope (Workhouse) Hospital, it was proved that the unfortunate woman threw herself from a third-storey ward window, "in the temporary absence of the nurse." The evidence adduced showed that the nurse in question had charge of three wards on three different floors, and the disaster could be in no way attributed to her negligence. The jury brought in a verdict of "Accidental death" (an accident which should have been impossible under proper management), and added three obvious recommendations—(1) That there should be a nurse on each floor; (2) that the nurses' hours on duty were too long; (3) and that the windows should be better secured. The case adds one to a long list of similar tragedies, due to the apathy and ignorance of Boards of Guardians, who will not provide proper attendance for the sick poor under their care.

### NURSES' ACCOMMODATION AT LAMBETH INFIRMARY.

At a recent adjourned meeting of the Lambeth Board of Guardians Mr. Mann strongly urged the need for a new nursing home, holding that the outlay of £2,000 or £3,000 would be well invested, and great convenience would accrue to the Board and the nursing staff. The resolution, "That the Guardians erect a building for the accommodation of a part of the nursing staff of the infirmary, if a site can be found for it, and that the Works Committee be asked to report upon the proposal," was adopted *nem. con.*, and the Chairman of the Works Committee expressed warm approval of the scheme, remarking that a fine site was available. He reminded the Board that an increased staff should be provided for.

### EXTENSIONS AT THE SWANSEA HOSPITAL.

At a recent special meeting of the Swansea Hospital Board of Management a report was received from a sub-committee appointed to consider, amongst other matters, the important question of additional accommodation for the nursing staff. Some difference of opinion arising with regard to the site of the proposed extension, the consideration of the matter was adjourned, on the motion of one of the governors, who thought it "too important a matter to be decided hastily, and by a small meeting." The Chairman announced a gift of £100 towards the cost of the new building from Miss Talbot.

### COTTAGE HOSPITAL BAZAAR AT CREWE.

Considerable excitement reigned at Crewe during the three days' bazaar lately held in Crewe Hall Park, and opened on the first day by H.R.H. Princess Louise and the Marquis of Lorne. Cheap tickets were issued for the occasion by the North-Western, North Staffordshire, and Great Western Railway Companies, and visitors thronged the town on the day of the opening, the weather being, fortunately, all that could be desired. Princess Louise and the Marquis of Lorne, who were staying at Crewe Hall, were accompanied by Lord Crewe and his three daughters; and the Hon. Mrs. Henniker, Sir Gerald and Lady Fitzgerald, and the Mayor and Mayoress (Alderman and Mrs. McNeill) and Mr. F. W. Webb were also present. The Mayor, in proposing a vote of thanks to the Princess for her presence, regretted the absence of two good friends to the Cottage Hospital—Mr. and Mrs. Henry Yates Thompson. Mr. F. W. Webb has been another generous donor to the institution, giving an additional subscription of £400 to enable the hospital to be handed over to the trustees free from debt, besides furnishing one of the wards.

### WANDSWORTH AND CLAPHAM UNION INFIRMARY.

At a meeting of the Wandsworth and Clapham Board of Guardians on August 6th a letter was read from Mr. Rogers, giving the following notice of motion: "To call attention to probationer nurses' wages, duties, nurses' quarters, meals, and hours, and move the following resolutions—'That the first year's wages of probationary nurses be increased to £14 per annum; that the board appoint six more nurses; that the work of cleaning brass, polishing handrails, and scrubbing balconies by probationary nurses be discontinued, and that such work be performed by the labourers; that an improved system of serving the nurses' food be adopted; that a full half-hour be allowed for breakfast, with an additional 15 minutes for bed-making, or that a dormitory maid be engaged for the purpose; that the Board proceed to consider the building of a new nurses' home immediately after the recess; that the medical superintendent be asked to draw up a scheme providing for three relays of nurses, no nurse to be on duty more than eight hours in twenty-four.'"

### NOTTINGHAM CONVALESCENT HOME.

A sale of work was held in the Wilford House grounds in aid especially of the Children's Branch of the Nottingham Convalescent Homes. The bazaar was opened by Lady Belper, and, in spite of very bad weather, a brisk sale was carried on. There is accommodation for twenty children in the Children's Home, which owes its establishment to the generosity of Colonel Seely.

### SHORT ITEMS.

The Hospital for Women, Soho Square, is closed entirely for repairs and alterations. It is to be re-opened early in September.—The case of Beatty *versus* Cullingworth was commenced in the Court of Queen's Bench on August 10th, and has now been adjourned until after the long vacation.—Nurse Larder, district nurse at Mosborough, was the other day presented by her many friends in the village, on the occasion of her marriage, with a handsome Queen Anne tea and coffee service, with oak tray.—The Grosvenor Hospital for Women and Children has received the sum of £50 from the Delmar Charitable Trust.

### Hygiene: For Nurses.

By John Glaister, M.D., F.F.P.S.G., D.P.H.Camb., Professor of Forensic Medicine and Public Health, St. Mungo's College, Glasgow, &c.

### XX.—SLEEP—HYPNOTISM—IDIOSYNCRASIES—IN RELATION TO HEALTH.

The state of sleep is characteristic of all animated nature, and a kindred phenomenon is observable in many plants. During this state the expenditure of nervous and muscular energy is repaired against a new period of work. With the ordinary phenomena of sleep all are practically acquainted, and they need not now be discussed. But there are certain attendant physiological conditions which are not generally understood. The predisposing causes of sleep may be summed up in the following, viz., (1) muscular or mental fatigue, or both; (2) a condition of quietness and rest; and (3) an attitude of muscular inactivity. Generally speaking, muscular fatigue can be repaired by resting the muscles during the waking condition. But the brain is in activity during the whole waking period. It therefore must be considered in two aspects, viz., (1) simply as one of the organs of the body, the waste of which requires to be repaired; and (2) as an organ having special and peculiar functions to perform, and which demands a special and peculiar mode of repair. Obviously, sleep has to do with the nutrition of the brain as a thinking and perceptive organ. During the whole period of wakefulness, whether in ideation, by emotions, sensations, impressions, or voluntary acts, through the channels of the sense organs, the brain is in constant activity. Hence in courting sleep we assume, as far as is possible, an attitude of body which is least liable to disturb the fading consciousness; thus we lie down, shut our eyes, and choose a quiet spot that our sense of hearing may not be kept active. Habit may modify these conditions, and man is able to adapt himself to conditions quite dissimilar from these mentioned. In going to sleep all of the senses do not go to sleep at the same time. Sight goes first in shutting our eyes, taste next, then smelling, then hearing, and last of all, the sense of touch. Neither does the body, as a whole, go to sleep at once. It begins at the points furthest from the centres of nerve control. It is for this reason that cold feet operate as a grievous hindrance to sleep. Certain physiological changes occur in the action of the heart, in the respiration, and in the bodily temperature. The number of heart-beats per minute is diminished; in like manner the respiratory action is less frequent; and a fall takes place in the bodily temperature usually about 1 deg. Fahr.

Causation of Sleep.—Many theories have been propounded regarding the proximate cause of sleep. We will briefly consider one or two of these. Since all theorists are agreed that the object of sleep is the recuperation of brain and nerve energy, an important factor is the condition of the blood-circulation of that organ. Round this several theories cluster. One of the very oldest and one of the most recent of these, consider that venous congestion of the brain is the cause. Another, which is supported by many observers, and by not a little experimental research, teaches that anæmia or comparative bloodlessness of the brain is the proximate factor. It is fairly certain the condition of the brain-circulation during sleep is one of anæmia, and from this and other facts, certain conclusions have been come to regarding the conditions of the brain blood-current which are most favourable to the onset of sleep. These are as follows: (1) A lessened flow of blood; (2) in consequence, a lessened supply of blood; and (3) an exhausted condition of brain-tissue. The first condition results from causes of a positional and functional kind; the second, from the physiological law of nutrition, that a resting organ demands less blood than one active; and the third is a periodic fact. Some years ago, Sir James Paget propounded the law of Rhythmic Nutrition, which simply is this, viz., that the nutrition of an organ, which has a rhythmic action, is accomplished in a special way to suit the special needs of the organ. Hence the heart is nourished in the "pauses" between the contractions. So with the brain. If we take a period of twenty-four hours, and call it a cycle, the brain rhythm would alternate between a condition of wakefulness and one of sleep—a condition of action and one of rest. During wakefulness the brain is the organ of mind, and of nerve-energy; during sleep it becomes merely an organ of the body, composed of so much nervous tissue-cells and fibres, having automatic control, however, over several bodily functions. During the period of active abeyance of function, the waste of the previous wakeful period is being repaired, until a moment of time arrives when some external stimulus reaches the brain through one of the organs of sense, and consciousness results.

Amount of Sleep Requisite for Health.—King Alfred's division of the day—"eight hours' work, eight hours' sleep, and eight hours' play"—so far as sleep is concerned, is an excellent average arrangement. One general principle may be laid down, viz., that the younger the person is, the more sleep is required. Infants sleep, in health, from eighteen to twenty of the twenty-four hours. Young children, up to the age of two-and-a-half years, require not less than fourteen hours daily; until school age, twelve hours; in adolescence, ten hours; and from that period till middle life, not less than eight hours. The aged sleep less than the young and middle-aged, but for them ten to twelve hours' daily rest in bed is beneficial in conserving their waning physical strength. The general reason for the longer periods of rest in those of tender years is that new growth as well as repair need to be attended to, whereas, as middle life is approached, it is only the daily waste that requires to be repaired. Many persons cultivate the habit of having less than eight hours daily for sleep. This may be borne with apparent impunity for a variably long period, but, in the end, evil effects are certain to follow. The brain worker, *a fortiori*, ought to secure eight hours' sleep daily, for it is of prime importance that the principal tool he uses should be bright, keen, and ready for work. Insomnia and general nervousness are too often the indications of inadequate brain rest or of mental worry. Both may be prevented, in large measure, by attention to the proper hours and periods of sleep, and by making every other engagement subordinate to these. The self-administration of hypnotic drugs ought to be strenuously resisted and opposed, and, at the earliest manifestation of sleeplessness, the advice of an experienced physician obtained. Not a single word can be said in commendation of the too prevalent modern habit which converts the night into the day at the expense of sleep. Such is fraught with inevitable mischief.

Hypnotism (Gr., *hypnos*—sleep) is the modern designation of psychical states, which formerly were known by the names of animal magnetism, electro-biology, Braidism, and Mesmerism, the last two from the surnames of men who practised and wrote concerning the art. Although the phenomenon has been known since the days of the Egyptians, and has been periodically investigated ever since, it is only within the last fifteen years that it has received the attention of scientific observers. Hypnotism is very difficult to define. It may be said to be a sleep-like condition induced by artificial means, but based upon physiological conditions of the brain, and which is wholly unconnected with any electro-biological or magnetic force. The precise form it will take in a person under its influence cannot be predicated. One person may assume a cataleptic condition, while another may be able to walk or perform complex movements at the will or suggestion of the operator. Public exhibitions of hypnotism are forbidden in Prussia, and ought to be prohibited in this country. It is a power which might be used by the irresponsible or criminal for purposes of gain or evil, and already hypnotic suggestion has been pleaded in France as a defence in a criminal charge. As a therapeutic agent it has been used beneficially, and, upon occasion, has taken the place of an anæsthetic in surgical operations. But in both respects it is still on its trial. For all these reasons, therefore, its practice ought to be confined to those who would be responsible to the State for its proper use. The principle of hypnotism is this—the concentration of the mental energy of the subject on an object, or an action, through the organ of vision, the other brain-centres being meanwhile inhibited, the production of fatigue in the visual centre—the only channel by which the person is conscious—and the induction of drowsiness and hypnotism, or sleep. There is more than this, however, for the operator is able to act on the will of the operated, thereby showing a degree of volition, but uncontrolled. There are certain psychic conditions allied to sleep and hypnotism which cannot be overlooked, such as dreaming, somnambulism, and catalepsy.

Dreaming comes on in sleep, and is characterised by some degree of mental activity, minus the government of the will; hence, dreams are usually of a distorted, irrational, and incoordinate character. Reverie is a dreamy condition in the waking state, and is produced by fixation or concentration of the mind on a certain object or train of thought, the person, meanwhile, being apparently and actually oblivious, in whole or in part, of external objects.

Somnambulism (Lat., *somnus*—sleep, *ambulo*—I walk), is a more complex condition. The subject of it may not only walk during sleep, but he may perform actions demanding complex co-ordination of muscles, skill, and, apparently, even calculation, all the time being unconscious to external influences. Somnambulism is a dream carried into action, the action being automatic and self-originated.

In all of these conditions the will-power is in abeyance, and they are usually found in healthy, but excitable persons.

Catalepsy is a condition wherein there is sudden suspension of volition, motion, and sensation, and is usually accompanied by a fixed condition of the limbs and trunk of the body in the position in which they were at the time of onset of attack. It is often allied with an abnormal condition of brain and nervous system, on the borderland, indeed, between health and disease.

Idiosyncrasies (Gr., *idios*—proper, *syn*—with, and *Krasis*—temperament).—An idiosyncrasy is something which is proper or peculiar to the temperament of the individual. It may be mental or physical. These peculiarities are not easily explicable from the physiological standpoint, but they have some relation to inherited conditions, or to impressions received in early childhood, which have left a lasting mark. They may exhibit themselves in the effects produced after partaking of certain substances, after the perceptions of certain odours, or by the irritation produced in the presence of certain conditions of atmosphere. Trousseau, in his classic work on clinical medicine, narrates cases of persons who were seized with acute asthma, one if he chanced to be in a bed-room when the feather-bed was being shaken, another, a chemist, if ipecacuanha was being powdered, or even dispensed, in his pharmacy, and a third—his own case—if he went into a room which had the odour of violets. Such cases are of nervous origin, and heredity has, doubtless, a large share in the production.

### Ethics of Nursing.

(*Continued from page* clxx.)

The Duty of the Nurse to the Public.

Sec. 1.—It is the duty of the trained nurse to take an active interest in the welfare of the community in which she lives, and to be on the alert whenever it lies in her power to assist in furthering public hygienic conditions. In the performance of her work she should always carry out strictly all measures adopted for the prevention of epidemic and infectious diseases, and as far as possible should induce those with whom she is brought in contact to follow her example. In the case of an epidemic, it is her duty to face the danger and to continue her labours for the alleviation of the suffering, even at the jeopardy of her own life.

Sec. 2.—A nurse should be willing to give a certain proportion of her time during the year to caring for the sick poor through the medium of district nursing associations.

The Duty of the Physician to the Nurse.

Sec. 1.—The physician should accord to a competent, trustworthy nurse his hearty loyalty and support.

Sec. 2.—If the services of a nurse are not satisfactory to the physician, and he deems it only just to inform her of the fact, he should refrain from doing so in the presence of the patient or of members of the family.

Sec. 3.—It is the duty of the physician to suggest, when necessary, that the nurse receives the proper amount of rest and relief from her duties, since only under these circumstances will she be able to do good work. Where the nurse has difficulty in obtaining the remuneration for her services which has been agreed upon, he should interest himself in seeing that justice is done.

The Duty of the Public to the Nurse.

Sec. 1.—The public should entertain a just appreciation of the qualifications necessary for those who undertake the responsibility of nursing the sick; they should discriminate between the legitimate claims of the scientific nurse and the assumptions of ignorant women; they should encourage and assist, by all means in their power, the higher development of schools for nurses, in order that the nursing throughout the country may be more uniform and reliable.

Etiquette.

I.—The cultivation of tact by the nurse in her dealings with those around her is absolutely necessary for the successful practice of the profession of nursing.

II.—When a nurse has been called to an urgent case, because the nurse engaged was not at hand, she ought, unless her assistance is still desired, to resign in favour of the latter immediately upon her arrival.

III.—When a nurse has been called to a patient of another nurse, in consequence of the sickness or absence of the latter, she ought, upon the recovery or return of the other nurse, and with the consent of the patient, to surrender the case.

IV.—When two or more nurses are on the same case, the nurse who has the case first should be considered the "head nurse," the others taking their orders from her and passing to her all orders received from the physician. They should never discuss the orders, or the methods of carrying them out, in the presence of the physician or of any member of the patient's family. When two or more nurses are caring for the same patient, the one first called is considered in charge, and she should have the right to remain with the patient when but one nurse is necessary.

V.—To obviate the necessity of questions, explanations, and discussions, which are often embarrassing and disagreeable to the nurse, a uniform fee should be decided upon by the Association of the Alumnæ and adhered to as closely as varying circumstances will permit.

Isabel Hampton Robb, Chairman.

Katharine de Long, Mary Heriot, Alice B. Conover } Committee.

### City of London Union Infirmary.

### LOCAL GOVERNMENT BOARD INQUIRY.

An inquiry, which occupied three days last week, and was continued on Tuesday, 18th, has been held at the City of London Union Infirmary by Dr. Downes, Inspector of the Local Government Board. Mr. Rose-Innes represented Dr. Buncombe, the medical superintendent, and Mr. Sherrington was the counsel for the matron, Miss Warburton.

It was shown by the Clerk to the Guardians that the infirmary is administered under the orders of the Local Government Board, dated December 2nd, 1874, and there do not appear to be any more recent orders relating to the medical superintendent, although additional regulations for the matron have been framed by the guardians. The medical officer was appointed in July, 1895, and succeeded his father, to whom he had previously acted as assistant for about five years. The matron was appointed in January of the present year, and entered on her duties on February 10th. A question from Dr. Downes as to whether the attention of these officers had been drawn to their duties was answered in the affirmative. The Clerk to the Guardians proceeded to explain that a scheme for the reorganisation of the nursing at the infirmary had been drawn out by the guardians, and had been finally sanctioned by the Local Government Board last December. It was decided to engage a trained matron, but, owing to lack of accommodation, the rest of the scheme had not been carried out as regarded the appointment of probationers, staff nurses, assistant matron, and night superintendent. On Dr. Downes asking whether any steps had yet been taken in the matter, he was informed that a contract would shortly be entered into to build.

Mr. Rose-Innes laid stress on the position of "supreme control" in which Dr. Buncombe was placed "as doctor and gentleman." The matron was superintendent of the female staff and responsible for the control and training of probationers. Mr. Rose-Innes referred to the matron's "non-obedience" to the medical officer, as destructive to the jurisdiction of that officer and interfering with his carrying out his duties impartially.

In reply to the matron's complaint that it was impossible for her to undertake the control and training of nurses if they were sent off floors without previous consultation with her, Dr. Buncombe explained that it had always been the custom to send a staff nurse to take patients to the asylum, to fetch children from the schools, and to accompany them when they were to be boarded out. In reply to an assertion that he had inspected certain cupboards since the present matron's appointment, Dr. Buncombe said that he had done so. When the matron first came she elected to go with him when he went round the wards, but she had not kept up the practice. The cupboards referred to were those in which food, not linen, was kept. He had had to complain that the patients' meals were not served punctually. He had gone to the kitchen and asked the assistant cook whether it was her fault or the matron's that the dinners were late. In answer to a question regarding the head nurse, Dr. Buncombe said that he considered her particularly careful and conscientious. She had had eighteen years' experience; some of her duties had been taken over by the matron. He was not aware that she "always acted independently of the matron"; if she had done so it was against his direct orders. The change of nurses on a certain Sunday had been made by him, as the matron was off duty for the day. He put a nurse he could trust in charge of the patients who were restless and bedridden—"bordering on imbecility." A new and inexperienced nurse was assisting the nurse, who was in temporary charge, and she committed an error of judgment by fastening a bandage across a patient. It was explained afterwards that the broad bandage, which was fastened also to the bedstead, was merely intended to prevent the patient removing the clothes, as she was very restless, and to keep her tidy whilst the doctor went round. The new nurse was ignorant of having erred, and was deeply distressed to find she was considered seriously to blame.

Mr. Sherrington enquired if Dr. Buncombe had begun to work in perfect harmony with Miss Warburton when she first took up her duties; to which he replied, "Perfectly." Friction was first caused by the matron making a report direct to the guardians on May 11th. She had reported a nurse to Dr. Buncombe about three times in April. With regard to the removal of nurses from the floor, he acknowledged that he did so at his discretion, without consulting the matron. Dr. Downes asked, "Whether Dr. Buncombe communicated with the matron about their transfer?" Dr. Buncombe replied, "Only through the nurse," and that he now wrote. He had sent for and reprimanded nurses without informing the matron. He did not recollect ever making any protest with regard to the duties which it was asserted that the matron had taken over from the head nurse.

Miss Warburton, in her evidence, gave information as to the previous appointments which she had held, and said that the complaints which she had entered in her report book had not been laid before the guardians, and considering this as a serious matter she reported direct to the committee. She had no book in which to report to the medical officer, but did so verbally. Asked if it would have been possible for other nurses to have been sent out on the duties for which experienced nurses were used, the matron replied: "It would seem to me better management to send someone else than to let a charge nurse go and leave a floor." On one occasion she had told Dr. Buncombe that she could not be responsible for the nursing if nurses were removed without her knowledge, and he had promised that it should not occur again. During June and July on various occasions nurses had been sent out by the medical officer. She considered that the cupboards should be left to her, and she was quite ready to hear any complaints which had to be made. She also said that the head nurse did not come for orders, and went out without her knowledge. The wrong-doing of nurses was not reported to her—the medical officer censured them himself and spoke to her about it afterwards. Dr. Buncombe found serious dissatisfaction amongst the nurses after his return from his holidays, and he went round the wards and questioned them.

Several nurses having been cross-examined on various points already brought forward, complaints of food were inquired into.

A dispenser considered the quality of the food bad and the cooking abominable. When asked for particulars, he said "Steak was swimming in fat." He had not complained to the matron.

The Steward's Clerk said he had complained of the food to the steward, who had referred him to the matron, and things had since improved.

The Day Porter gave evidence as to the rule for nurses' passes. He received instructions when he came that the nurses' passes would be signed by the matron. The medical officer had latterly informed him that no nurse should go out unless her pass was signed both by matron and medical officer.

On one or two occasions nurses had gone out with passes signed by medical officer only, and also on a verbal order. In reply to an assertion that a nurse's leave had been stopped by the matron, it was shown that the nurse had refused to go out in the evening, as the medical officer had said she was to have time every afternoon.

In the course of the inquiry, Mr. Sherrington asked a question with regard to the resignation of twenty-five nurses in the year which preceded Miss Warburton's appointment, and stated that some discussion having taken place on the subject, the medical officer had written to the clerk of the Guardians and suggested adequate reasons for the resignations, as there had been a report that they "had been driven out by his harsh treatment." Mr. Sherrington explained that he had referred to this correspondence merely to show that resignations amongst the nursing staff had taken place before, as well as since, the appointment of the matron.

The inquiry was continued throughout Tuesday, and after a large amount of evidence heard on both sides, of more or less the same character as that which we have reported, Dr. Downes was thanked for the impartiality which he had exhibited throughout the proceedings. His report will in due course be handed to the Local Government Board. During the hearing of the case, the Medical Superintendent of the Infirmary brought forward thirty-six complaints, the Matron having many counter charges, the whole of the evidence going to show that the authority of the two officers was not strictly defined, and constant friction on small points had ultimately brought matters to the present *impass.*

### Holidays and Health.

[Readers of The Hospital in need of information about health resorts at home or abroad, or desirous of aid in forming holiday plans, are invited to send queries to Editor, 428, Strand, W.C. (marked "Travel" on outside of envelope), which will be answered under this section.]

### ALDEBURGH-ON-SEA.

Aldeburgh is a charming little seaside village on the Suffolk coast, and those in search of a pleasant, quiet, and breezy place, within three or four hours of London, cannot do better than spend a few weeks there. Lodgings are plentiful, if not particularly luxurious, and there are two excellent boarding-houses, one of them, Hill House, is owned by Mrs. Thornbury, and Mrs. Jay keeps a private hotel and boarding-house. The first of these, which, by the by, calls itself a "Home for Paying Guests," is kept by a connection of the well-known Garrett family, who have done so much to improve their native place. It is a charming house to stay in, and in all ways thoroughly comfortable, "its very wall papers and decorations being a distinct rest and pleasure," as one of its inmates was heard to remark, and it is by no means expensive.

Formerly Aldeburgh was large and important, but the North Sea has from time to time washed away so much of it that it now looks only like a small village, and so recently as two winters ago the houses on the low beach were almost swept away, some of them only being saved by opening both their back and front doors and letting the sea pour through them. A memorial of this combined high tide and high wind remains in banks of shingle that were washed up from the bed of the sea, and that now form a kind of raised beach at a considerable distance inland, which serves as a bank for the little river Alde, which flows almost parallel with the sea for about five miles.

There is excellent bathing; the waves are not too rough to spoil the pleasure of even a moderate swimmer, and the boating is delightful; there are plenty of boats for hire, and a whole day may be spent most enjoyably out on the sea or on the river, for a small sum.

Excursion steamers, donkeys, bands, and niggers are unknown, and to this fact Aldeburgh owes much of its quiet restfulness, for trippers do not consider it worth coming to, and the place consequently possesses a good deal of the charm, repose, and respectability of Cromer, with the additional advantage of being less expensive.

On the beach stands a most curious specimen of 12th century architecture, the Mote House, or ancient town hall, a half-timber building, with beautifully carved bargeboards, and an outside wooden staircase; quite an unique erection, and an excellent subject for sketches or photographs.

The parish church is a fine old building, and contains a memorial to the poet Crabbe, who was a native of the place, and also one to Professor Fawcett, that 19th century hero who did so much good and useful work in spite of his terrible affliction.

Several charming expeditions can be made from Aldeburgh. Leiston is within an easy walk or a short train journey, and there very fine ruins of an ancient Benedictine abbey are to be seen. They are now sadly neglected, the chancel of the abbey being used for a cowshed, while one of its beautiful transepts is roofed in to form a barn! For the place belongs to a farmhouse, the owners of which evidently consider it a merit to utilise all the walls they can, merely considering them as convenient walls and angles, and further, nothing!

In quite another direction is Orford Castle, a magnificent Norman tower not unlike Rochester Castle. It can be seen for many miles out at sea, and when George II. was overtaken by a storm in the North Sea on one of his journeys from Hanover, he landed at Orford Ness and gladly took refuge in the old tower. From its roof there is a very extensive view; the country round is singularly flat, so that the eye seems to travel on uninterruptedly to the "edge of creation" both on the land as well as on the sea side—an effect that one rarely experiences out of Holland. The sensation that this flat country has upon the beholder is restful and pleasant.

For those, indeed, who from choice or necessity take their ordinary life at a gallop, it is a distinct advantage to take their holiday at a slower pace—and an effect of slowness and breadth will quickly makes itself felt upon the high-strung holiday-seeker, if he will only fall into the spirit of the place and thereby reap the benefits.

It is a pleasant sail down the river from Aldeburgh to Orford, and on arriving there is a capital little inn not far from the Castle, where an excellent tea can be had, if we wait for the cows to be milked—and of course we do wait. Orford church is rather remarkable, some of its ruined pillars being as beautiful as the famous "Prentice pillar" in Roslyn Chapel. Aldeburgh has good golf links a little way inland, and the visitor is allowed to play there on payment of a by no means prohibitive subscription. Everything, indeed, is done by the authorities of the place to render a visitor's stay there pleasant. The railway fare from Liverpool-street, third-class, is 8s. 4½d., or cheap return for fifteen days, 13s. The journey takes about three hours.

### Appointments.

### MATRONS.

Omagh District Asylum.—Miss Margaret E. McFarland has been appointed Matron of this institution. She received her training at the Tyrone Infirmary, where she afterwards held the position of head nurse.

### Minor Appointments.

Keighley Union Infirmary.—Miss Ann Bates has been appointed head nurse at this infirmary. She received her training at the Leeds General Infirmary, then becoming charge nurse at the Beverley Cottage Hospital, afterwards holding the appointment of matron at the Cottage Hospital, Chalfont St. Peter, night superintendent at Whitechapel Infirmary, and head nurse at Steyning Union Infirmary, Shoreham.

Warwick Workhouse Hospital.—Miss Sabina Wright has been appointed Head Nurse at this infirmary. Particulars of training are not forthcoming. Miss Wright has acted as nurse and as charge nurse at the West Riding Asylum, Wakefield, and as charge nurse at Hunslet Union Infirmary, Leeds.

Cheshire County Asylum, Macclesfield.—Miss Haslewood asks us to correct the statement that she has been appointed head nurse at the above institution. Miss Haslewood remains for the present at the Hospital for Consumption, Brompton, where she holds the position of night superintendent.

Oswestry and Ellesmere Cottage Hospital.—Miss Helen Scott has been appointed matron at this hospital. She was trained at Addenbrookes, Cambridge, and for the last four years has held the post of sister of the male wards at the Dorset County Hospital. Miss Scott has excellent testimonials, and on leaving was presented with a case of silver spoons from the matron, and with several other gifts from the nursing staff and servants. She takes all good wishes to her new appointment.

### Dress and Uniforms.

By a Matron and Superintendent of Nurses.

### THE ALFRED HOSPITAL, MELBOURNE.

The colonies are so far away that perhaps few of us realise the enormous advance they have made in every way of recent years. The trained nurse occupies her own little niche in the progressive movement, and, as shown in our illustration, is no way behind her sisters over here. The Alfred Hospital nursing staff has for behind and fasten with buttons on to the band. The pretty mob-shaped cap is made of muslin, embellished round the edge with a gophered lace frill. White cuffs and collars make a becoming finish to the neck and wrists, the general effect being further enhanced by a white armlet worm round the left arm, on which a Maltese cross in red appears. The pupil nurse's costume is lilac haircord, which has a very cool,

PUPIL NURSE SISTER

long held a prominent position in Melbourne, and ranks among the most enlightened and highly-trained in Australia. The sisters, one of whom appears in the sketch before us, wear a dress of small blue-and-white check, made quite plain and full in the skirt, which is attached to the bodice by a band at the waist. A large white linen apron covers the dress, and the bib is finished off with straps which cross fresh appearance, and as such eminently adapted for wear in a sick ward. The cap and apron are similar in shape and design to those worn by the sisters. They also wear a white armlet round the arm, but the cross is worked in outline only, instead of an entire filling of red. White linen cuffs and collars complete what may justly be described as a very attractive costume.

### For Reading to the Sick.

**Motto.**

"Fret not thyself."—*Ps.* xxxvii. 1.

**Verses.**

Perplexities do throng upon my sight,
Like scudding fog-banks, to obscure the light;
Some new dilemma rises every day,
And I can only shut my eyes and pray.

Lord, I am not sufficient for these things;
Give me the light that Thy sweet presence brings;
Give me Thy grace, give me Thy constant strength,
Lord, for my comfort now appear at length.

It may be that my way doth seem confused,
Because my heart of Thy way is afraid;
Because my eyes have constantly refused
To see the only opening Thou hast made.

And now I pray Thee, Lord, to lead Thy child—
Poor wretched wanderer from Thy grace and love;
Whatever way Thou pleasest through the wild,
So it but take her to Thy home above.

—*Christian Lyrics.*

Say, did impatience first impel
Thy heaven-sent bond to break?
Or could'st thou bear its hindrance well,
Loitering for Jesu's sake?

Oh, might we know! for sore we feel
The languor of delay,
When sickness lets our fainter zeal
Or foes block up the way.

Lord! Who thy thousand years dost wait
To work the thousandth part
Of thy vast plan, for us create
With zeal, a patient heart.

—*Newman.*

**Reading.**

Our anger and impatience often prove much more mischievous than the things about which we are angry or impatient.—*Marcus Aurelius.*

"A most unhappy perturbation of mind is created among all those, of every rank, whose tempers are contrary to the holy mind of Christ. Even heaven itself would bear the semblance of hell, could such minds surround the throne of God; for perfect holiness and infinite happiness are inseparably united."—*Rev. Rowland Hill.*

Many things in this life do fall out as we would not. Give me strength to resist, patience to endure, and constancy to persevere.

Fret not thyself—is it any use to read this Psalm when you are racked with a continual fever of fretting and nerves, and anxieties, half of which you know (but cannot feel) to be groundless? Our Lord once took a woman's hand and "the fever left her." If He took your hand, this worse fever would leave you—this fever of the nerves which is wearing you to threads. Is it your fault, or His, that He does not do so? The woman "arose and ministered to them." How you envy her! But it is not your bodily illness which hinders you from ministering half so much as that spiritual fever of a rebellious will. . . . It is indeed putting "matter in the wrong place," if you imagine that any physical cause need so quench the Spirit as to put any one of His gifts entirely out of your reach. It may be that, to the end, you will sigh over "that hardest of all precepts, to rejoice," and in your garden peace may flourish less if you are of those who "bring forth new fruit according to the months, because their waters they issued out of the Sanctuary."—*Miss Lucy Soulsby.*

### Royal British Nurses' Association.

WAS THE LETTER REGISTERED?

We have received the following communication from Miss G. E. Guiseppi, acting secretary of the Royal British Nurses' Association, in reference to a paragraph under the above heading in The Hospital of August 1st:—

Sir,—With reference to a paragraph which appeared in The Hospital of the 1st inst. as to whether a certain letter forwarded to the Royal British Nurses' Association by Miss Breay was registered or not, we have been in further correspondence with the Post Office upon the matter, and have received from them the following reply:—

"General Post Office, London, 15 August, 1896.

"Madam,—With reference to your letter of the 6th instant, I am desired to inform you that the reply sent to Miss Breay, of which you enclosed a copy, relative to the envelope which is returned herewith, was quite correct. The letter in question was both registered and sent as an express letter, as indeed is shown by the counterfoil of the receipt which you signed. but the officer who accepted the letter at the Vere Street Post Office unfortunately omitted, through an oversight, to mark the cover with blue lines in the usual manner, an omission which led to the wrong information subsequently given to you verbally at that office. Due notice has been taken of the irregularity, which is regretted, as also is the unnecessary trouble which has been occasioned to you in the matter.—I am, madam, your obedient servant,

"Miss E. G. E. Giuseppi." "J. Swainson.

I may state that, previous to receiving this letter, we had been informed by the officials at Vere Street, who saw the cover which enclosed the letter in question, that it was certainly not that of a registered letter.—I am, Sir, faithfully yours,

Eugenie G. E. Giuseppi,
Acting Secretary.

### Notes and Queries.

**Queries.**

(146) *Nurses' Co-operation.*—Is there any other private nursing institution in London, besides that in New Cavendish Street, which is conducted on really co-operative principles? I have answered advertisements from several so-called "Co-operations" and find on inquiry that they are purely personal speculations, and co-operative only in name.—*Veritas.*

(147) *Epilepsy.*—Please tell me if there is any institution where hopeless cases of epilepsy can be not only treated, but permanently cared for.—*Caritus.*

(148) *Sanitary Inspectorship.*—How should I set about training for sanitary inspecting work; and can you tell me anything about the cost of training, and recommend books for study?—*Nurse A. D.*

(149) *Dispensing.*—Kindly give me the names of one or two books to study in preparation working for the assistants' diploma of the Apothecaries' Hall? Is the exam. a difficult one?—*F. C.*

(150) *Enema Clip.*—Kindly tell me where I can obtain the clip for holding enema syringe to basin, advertised in The Hospital some time since.—*Nurse F.*

(151) *Rome.*—Please tell me if there is a home in Rome for private nurses. I find St. Paul's Home, Via Palestro, is permanently closed, and I can hear of no other.—*Italy.*

**Answers.**

(146) *Nurses' Co-operation* (*Veritas*).—Your experience is that of most nurses, and before joining any so-called "Co-operation" *most* careful inquiries should be made. You will find a list of institutions in Burdett's "Hospitals and Charities," and your best plan is to write for all particulars to those which seem to answer your requirements, taking care to obtain full information before entering upon any engagement.

(147) *Epilepsy* (*Caritus*).—There is the Meath Home of Comfort for Epileptics (women and girls) at Westbrook, Godalming, and the Colony for Epileptics at Chalfont St. Peter, Bucks, at present for men only. The women's home is in course of construction. Write to the Secretary, 12, Buckingham Street. Strand. Payment is required.

(148) *Enema Clip.*—The makers of the clip about which you ask are Messrs. Reynolds and Branson, Briggate, Leeds.

(149) *Sanitary Inspectorship* (*Nurse A. D.*).—Write to Miss Lamport, 52, St. John's Wood Road, N.W., who will give you all the information you wish for.

(150) *Dispensing F. C.*).—The examination of the Society of Apothecaries is not a difficult one, but a thorough knowledge of materia medica, as contained in the "British Pharmacopœia," is necessary. In Burdett's "Hospital Annual" for 1894 you will find a useful chapter on this subject, with full directions and lists of books, amongst them the following: Morris's "Class Book of Inorganic Chemistry" (Philip and Son Fleet Street, 2s 6d.), Bentley's "Materia Medica" (Longmans, 7s. 6d.), and "British Pharmacopœia" (Spottiswoode, 6s.). The "Year Book of Pharmacy" might give you some useful information.

(151) *Rome* (*Italy*).—We do not know of any other Home save the one to which you have written.

## Figures and Tables

**Figure f1:**